# How Epstein–Barr Virus Drives Multiple Sclerosis: New Mechanisms and Therapeutic Lessons

**DOI:** 10.1002/mco2.70835

**Published:** 2026-06-19

**Authors:** Dan Liu, Wenhui Fan, Pengtao Jiao

**Affiliations:** ^1^ Institute of Animal Science Chinese Academy of Agricultural Sciences Beijing China; ^2^ College of Veterinary Medicine Northwest A&F University Yangling China; ^3^ College of Chemistry and Molecular Sciences Henan University Kaifeng China

1

Recently, three Cell articles elucidated how Epstein–Barr virus (EBV) triggers multiple sclerosis (MS) pathogenesis, exploring T‐cell cross‐reactivity, EBV‐induced B‐cell dysfunction, and synergistic genetic‐environmental interactions [1, 2, 3]. These findings provide a framework to enhance understanding of MS pathogenesis and guide therapeutic development.

MS is an autoimmune disorder characterized by inflammation, demyelination, and neurodegeneration within the central nervous system (CNS) (Figure 1a). Globally, around 2.8 million individuals are estimated to be affected by this condition, with EBV infection recognized as the most significant environmental risk factor. Indeed, nearly all MS patients are seropositive for EBV, and following infection, this virus can establish lifelong latency within memory B cells. Previous research has suggested that EBV‐induced alterations in the B‐cell transcriptome could contribute to MS pathogenesis. However, the underlying molecular mechanisms are yet to be fully elucidated.

**FIGURE 1:**
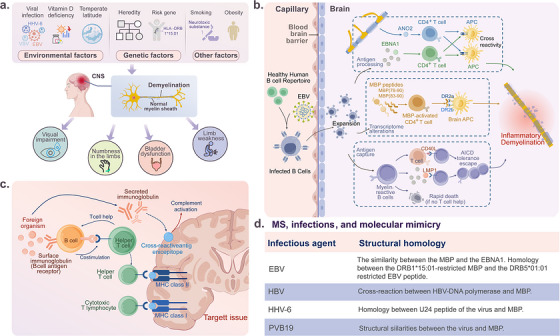
Mechanisms of infection‐associated autoimmunity in multiple sclerosis (MS). Created with BioGDP.com. Summary of the key etiological factors and common clinical manifestations of MS. The predisposing factors include genetic susceptibility (e.g., HLA haplotypes), environmental triggers (with Epstein–Barr virus [EBV] infection being the most prominent), and lifestyle factors. The major clinical symptoms arising from central nervous system (CNS) demyelination include visual impairment, limb numbness, and muscle weakness. Schematic illustrating pathways through which EBV infection may contribute to MS pathogenesis. Three key processes are highlighted: (1) Molecular mimicry whereby EBV antigens, such as EBNA1, activate cross‐reactive T cells that subsequently target CNS self‐antigens (e.g., anoctamin‐2 [ANO2]). (2) B‐cell reprogramming where latent EBV infection in genetically predisposed individuals (e.g., HLA‐DR15 carriers) alters B‐cell function, leading to the aberrant presentation of myelin autoantigens, such as myelin basic protein (MBP). (3) B‐cell survival signaling in which the EBV LMP1 protein mimics CD40 signaling within the brain, thereby providing essential survival and activation signals that promote the persistence and pro‐inflammatory activity of autoreactive B cells. Schematic representation of the molecular mimicry mechanism showing how an immune response targeting an infectious agent may be erroneously redirected against self‐tissues. Pathogen‐derived peptides (depicted as orange spheres) share structural similarity with self‐peptides (depicted as blue spheres), enabling cross‐reactive antigen presentation and T‐cell activation. These activated T cells may subsequently assist B cells in producing autoantibodies or directly mediate tissue injury, thereby initiating autoimmune responses. (d) Overview of infectious agents associated with MS. The table lists various pathogens (e.g., EBV and hepatitis B virus) and highlights their potential to harbor antigens that may cross‐react with CNS components.

In addition, the HLA‐DR15 haplotype represents the strongest known genetic risk factor for MS, accounting for up to 60% of inherited susceptibility. This haplotype carries the HLA‐DRB1 and HLA‐DRB5 genes, which encode the MHC class II molecules DR2a and DR2b, respectively. These molecules are primarily responsible for presenting antigenic peptides to CD4^+^ T cells, thus being consistent with the characteristics of MS as a CD4^+^ T‐cell‐mediated autoimmune disorder. Other risk alleles, such as IL2RA and IL7R, have also been implicated and may modulate T‐cell responses to EBV infection, further supporting a gene–environment interplay. Early research has demonstrated that EBV infection and HLA‐DR15 can synergistically activate CD4^+^ T cells capable of cross‐reacting with self‐antigens through molecular mimicry, thereby initiating autoimmune responses [4]. Yet, the precise mechanism by which EBV‐driven B‐cell dysfunction, especially as a result of extensive transcriptomic changes, interacts with genetic susceptibility to drive the full molecular pathogenesis of MS remains a critical unresolved question.

Thomas et al. [1] have provided direct evidence of T‐cell cross‐reactivity between the EBV nuclear antigen EBNA1 and the CNS autoantigen ANO2. Specifically, they detected significant T‐cell responses to a homologous region shared by ANO2 (residues 79–168) in peripheral blood mononuclear cells (PBMCs) from over 57% of untreated MS patients and natalizumab‑treated MS patients, with significantly higher reactivity observed compared with that of healthy controls. Furthermore, in SJL/J mice, immunization with ANO2 or EBNA1 could induce robust cross‐reactive CD4^+^ T‐cell and antibody responses, while preimmunization with ANO2 markedly increased disease severity and relapse frequency in experimental autoimmune encephalomyelitis (EAE). Mechanistically, T‐cell clones derived from HLA‐DRB1*15:01‐positive patients were able to recognize both antigens, while single‐cell TCR sequencing identified 55 shared amplified clones. Overall, those findings provided molecular‐level validation for the molecular mimicry hypothesis, yet the prevalence of ANO2‐specific T cells in treatment‐naive and broader MS populations remains to be elucidated (Figure 1b,c).

EBV infection can also promote autoimmune responses against the myelin sheath by altering the antigen‐presenting function of B cells. Through flow cytometric analysis of HLA‐DR15 surface expression and mass spectrometry‑based immunopeptidomics to identify peptides eluted from HLA‑DR15 molecules, Wang et al. [2] demonstrated that EBV‐infected B cells not only exhibited enhanced expression of HLA‐DR15, a molecule associated with MS risk, but also presented myelin basic protein (MBP) autopeptides (MBP_78–90_ and MBP_83–90_) that are typically absent from healthy B cells. Using CFSE dilution and HLA‐DR blocking assays with CD45RA‐PBMCs, they further showed that these peptides were then selectively recognized by CD4^+^ T cells derived from both the peripheral blood and cerebrospinal fluid of patients with MS. Importantly, since these F90‐terminal MBP peptides are not present in the thymus, the corresponding autoreactive T cells that recognize them can escape negative selection, remain quiescent in the periphery, and persist until activated by EBV‐associated infection events (Figure 1b,c). Although this study provides the first immunopeptidome‐level link between EBV infection and MBP autoantigen presentation, further validation in more physiological primary‐infection models and non‑transformed B cells is warranted.

In addition, the survival and pathogenicity of autoreactive B cells within the CNS depend on sustained co‐stimulatory signals. For instance, Kim et al. [3] showed that autoreactive B cells infiltrating the brain were able to capture myelin oligodendrocyte glycoprotein (MOG) and present it locally. However, their survival was strictly dependent on CD40 signaling, with a loss of this signal triggering apoptosis. Crucially, by transgenic expression of the EBV latent membrane protein 1 (LMP1) in mouse MOG‐specific B cells, the viral protein functionally mimicked CD40 co‐stimulation, thereby rescuing these B cells and synergizing with B‐cell receptor (BCR) signaling to promote their proliferation. In vivo B‑cell‐mediated pathogenic model in mice, this mechanism was further shown to directly induce focal demyelinating lesions, along with complement deposition and T‐cell infiltration. Overall, these findings establish a direct link between EBV infection, aberrant B‐cell activation, and CNS‐specific tissue damage, thus elucidating the mechanism through which environmental factors and immune dysregulation collectively drive the pathogenesis of MS (Figure 1b,c).

In conclusion, the above studies provide a coherent mechanistic framework that links EBV infection to MS pathogenesis while clarifying its synergistic interaction with the main genetic risk factor, HLA‐DR15. Following EBV infection, genetically susceptible individuals harbor the virus within B cells, and this condition not only promotes the activation of cross‐reactive CD4^+^ T cells that target self‐antigens (e.g., ANO2) via molecular mimicry but also reprograms B cells to aberrantly present myelin autoantigens (e.g., MBP). Moreover, when the blood–brain barrier is compromised due to infection or trauma, these dysfunctional T and B cells might infiltrate the CNS. Subsequently, within the brain, LMP1‐mediated CD40 mimicry provides essential survival and activation signals to autoreactive B cells, thereby preventing their elimination and sustaining inflammation and antibody‐mediated injury. Such coordinated actions of autoreactive T and B cells ultimately drive myelin destruction and neurodegeneration, the underlying pathological substrates of the clinical manifestations of MS. It should be noted, however, that while these pathways are supported by the studies discussed, definitive proof that they drive disease in humans is still lacking and requires further investigation.

These insights carry significant translational significance. Indeed, from a diagnostic perspective, the detection of EBNA1/ANO2 cross‐reactive T cells in peripheral blood, T cells that are responsive to aberrantly presented MBP peptides or LMP1‐presenting B cells may serve as novel biomarkers for early diagnosis or prognostic assessment of MS. Furthermore, the pathogenesis of MS is intricate, influenced by various factors, such as genetic predispositions, environmental elements and immune dysregulation (Figure 1a). Thus, targeting EBNA1/ANO2‐specific cross‐reactive T cells, inhibiting LMP1‐mediated signaling pathways and selectively eliminating pathogenic B‐cell subsets may provide a highly promising strategy for precision‐based interventions. Finally, even though EBV currently represents the most compelling infectious contributor to MS, other viruses, such as HBV, HHV‐6, and PVB19, may also influence disease development through related or distinct mechanisms [5] (Figure 1d). Beyond implicating EBV as a major environmental trigger, the findings presented above offer a broader conceptual framework for understanding how diverse infectious agents could be involved in the complex pathogenic network underlying MS, pending validation in future studies.

## Author Contributions

P.J. designed the project. D.L. and W.F. wrote and revised the manuscript. D.L. drew the figure. All authors have read and approved the final manuscript.

## Ethics Statement

The authors have nothing to report.

## Conflicts of Interest

The authors declare no conflicts of interest.

## Data Availability

The authors have nothing to report.
